# Supplementary material for: The Older the Better: Infanticide Is Age-Related for Both Victims and Perpetrators in Captive Long-Tailed Macaques
Source: Biology (Basel). 2022 Jul 4;11(7):1008. doi: 10.3390/biology11071008 (PMC9311617; doi:10.3390/biology11071008)
Supplement: Supplementary file 1 [file biology-11-01008-s001.zip › biology-1770413-supplementary.pdf]

Table S1. Infant and mother characteristics. Characteristics of victims of infanticide (K) and infants at risk of infanticide that were not killed (NK) and their mothers during infanticidal male takeovers. Infants from an age between -162 (start pregnancy) and 215 (maximum age to shorten IBI) days of age were considered to be at risk of infanticide.

Infant characteristics					Mother characteristics			
**Name**	**Fate**	**Sex**	**Age at start male tenure (days)**	**Age at death (days)**	**Name**	**Age at start male tenure (years)**	**Experience**	**Rank**
Pooh2[a]	K	M	248	248	Ola	7.5	yes	low
Nnm1[b,c]	K	M	-1205[b,c]	60	Deha	0.5	no	low
Nnf1	K	F	-75	62	Papoa	8.6	yes	high
Het Loo[b]	K	M	-270 [b]	112	Orka	9.3	yes	low
Nnf2	K	F	32	37	Henna	12.9	yes	low
Nnm2	K	M	-39	26	Lixa	17.5	yes	high
Kahlua	K	F	-47	94	Uha	3.7	no	low
Veenema[a]	K	F	404	486	Milva	4.9	no	low
Bapao[b,d]	K	M	176[b]	197	Alfa	8.2	yes	high
Haifa	K	F	31	132	Fokwa	7.6	yes	low
Nnm3[b]	K	M	-194[b]	2	Sea	8.3	yes	low
Balata	K	F	141	147	Tres-bella	4.6	no	low
Lao	K	M	20	89	Alfa	18.5	yes	high
Tequilla[b]	K	F	-182[b]	87	Lambada	7.1	yes	high
Manuchao	K	M	-58	69	Anastasia	3.9	no	high
Spa	NK	F	122		Pukkie	16.0	yes	low
Deha	NK	F	177		Haasje	9.6	yes	low
Groucho	NK	M	151		Henna	7.0	yes	low
Alfa	NK	F	149		Felix	14.6	yes	high
Tao	NK	M	149		Abba	4.6	no	low
Sla	NK	F	114		Lea	4.9	yes	high
Bona	NK	F	77		Nasa	5.5	no	low
Ikea	NK	F	33		Exa	12.6	yes	high
Livorno	NK	M	20		Norma	11.6	yes	low
Aloa	NK	F	-72		Oor	13.6	yes	high
Nena	NK	F	210		Nasa	10.2	yes	low
Miao	NK	M	128		Abba	9.3	yes	low
Djoba	NK	F	125		Bona	4.9	no	low
Sjattoa	NK	F	123		Opa	4.1	no	high
Bilboa	NK	F	106		Orka	9.3	yes	low
Hanso	NK	M	106		Saga	9.5	yes	low
Epha	NK	F	36		Henna	11.7	yes	low
Wubbo	NK	M	-33		Boa	11.7	yes	high
Mikado	NK	M	195		Dracula	5.3	yes	high
Nononono	NK	M	190		Nasa	11.5	yes	low
Paramo	NK	M	169		Manna	11.0	yes	low

Table S1. *Cont.*

Infant characteristics					Mother characteristics			
**Name**	**Fate**	**Sex**	**Age at start male tenure (days)**	**Age at death (days)**	**Name**	**Age at start male tenure (years)**	**Experience**	**Rank**
Toco	NK	M	167		Cheba	5.5	yes	high
Esso	NK	M	141		Geel	16.5	yes	low
Kufo	NK	M	-39		Felix	21.5	yes	high
Rastafa	NK	F	201		Freya	5.4	no	low
Jura	NK	F	176		Roza	13.4	yes	low
Wiepkema	NK	F	173		Milva	5.8	no	low
Katua	NK	F	105		Upupa	8.4	yes	high
Stoa	NK	F	72		Ola	19.7	yes	low
Bolero	NK	M	63		Rassoa	4.2	no	low
Nacho	NK	M	28		Hoempa	13.0	yes	high
Inca	NK	F	-36		Chiwawa	3.6	no	low
Zazaa	NK	F	-54		Aha	9.1	yes	high
Winnebago	NK	M	-27		Geisha	8.8	yes	low
Mustafo	NK	M	-140		Fransisca	4.1	no	low

[a,b] Infants that were killed, but do not meet the criteria of being at risk since they were: [a] either too old to shorten the IBB, or [b] could have been fathered by the infanticidal male himself since he attained the alpha position before the time of conception. [c] This infant was killed about three years after the alpha male became resident in the group. [d] Infant that was killed during a reintroduction of the alpha male after an absence of eight months, he was sired during the previous tenure of the same male. K = killed, NK = not killed, M = male, F = female.
